# Genetic variation and genetic structure of five Chinese indigenous pig populations in Jiangsu Province revealed by sequencing data

**DOI:** 10.1111/age.12560

**Published:** 2017-05-22

**Authors:** Q. Xiao, Z. Zhang, H. Sun, H. Yang, M. Xue, X. Liu, W. Zhang, Y. Zhen, M. Zhu, Q. Wang, Y. Pan

**Affiliations:** ^1^ Department of Animal Science School of Agriculture and Biology Shanghai Jiao Tong University Shanghai 200240 China; ^2^ National Station of Animal Husbandry Beijing 100125 China; ^3^ Jiangshu Station of Animal Husbandry Nanjing 210036 China; ^4^ Shanghai Key Laboratory of Veterinary Biotechnology Shanghai 200240 China

**Keywords:** genome sequencing, GGRS, Indels, SNPs

## Abstract

In this study, we investigated the genetic variants, including SNPs and indels (short insertions or deletions, less than 50 bp in length), in the genomes and genetic structures of five pig populations (in the northern Taihu Lake region, Jiangsu Province) using the genotyping by genome reducing and sequencing (GGRS) approach. A total of 581 million good reads with an average depth of 11× and an average coverage of 2.16% were used to call variants. In general, 202 106 SNPs and 34 415 indels were obtained, of which 2690 SNPs and 224 indels were capable of inducing protein‐coding changes. The genes containing these variants were extracted for functional annotation. The results of gene enrichment analysis revealed that the SNPs under investigation may be associated with reproduction, disease resistance, meat quality and adipose tissue traits, whereas the indels were associated mainly with adipose tissue and disease. Analysis of the genetic structure showed that each population displayed comparable, large differentiations from the others, indicating their uniqueness. In conclusion, the results of our study provide the first genomic overview of the genetic variants and population structures of five Chinese indigenous pig populations.

Jiangsu Province, in which the giant lake Taihu is located, lies in the eastern part of China. Its favourable geographical and social conditions have given rise to resourceful livestock breeds. Some Taihu pig breeds, such as Meishan, Erhualian and Mi—well‐known prolific breeds of the world (Zhang 1986)—are distributed throughout the Jiangsu Province. Apart from these breeds, other pig populations, including Jiangquhai, Dongchuan, Huaibei, Shanzhu and Hongdenglong, are also distributed throughout the province (Fig. S1). These populations perform well for many economic traits, such as reproduction, adaptability, disease resistance and meat quality (China National Commission of Animal Genetic Resources 2011). To explore the genetic mechanisms underlying these desirable qualities, the genetic variants harboured by these five pig populations should be identified. The declining sizes of these five populations, caused by the importation of exotic commercial pig breeds, are a reminder that the conservation and utilization of these genetic resources are crucial. Therefore, the detection of genome‐wide genetic variants and exploration of the genetic structure of the five populations in Jiangsu Province are necessary. The aim of this study was to identify and annotate genetic variants (including SNPs and indels less than 50 bp in length) and investigate the population structure based on SNP data to evaluate the conservation and utilization of these genetic resources.

A total of 129 samples from five pig populations were collected from conservation pig farms in Jiangsu (Table 1). The DNA samples were genotyped using the genotyping by genome reducing and sequencing (GGRS) protocol (http://klab.sjtu.edu.cn/GGRS/) (Chen *et al*. 2013) (Appendix S1). A total of 581 401 615 good reads with an average depth of 11× and an average coverage of 2.16% were generated (Table 1). The average quality score for each base was at least 20 (Fig. S2a and b). The reads for each population ranged from 1.37 million (Dongchuan) to 5.83 million (Hongdenglong) (Fig. S2c). The SNP calling was performed using samtools software (version 0.1.19) (Li *et al*. 2009) and the unified caller of the genome analysis toolkit (gatk) (McKenna *et al*. 2010). The final results were obtained from the overlapping data of these two methods. Missing genotypes of SNPs were imputed with stitch (Davies *et al*. 2016). To ensure detection accuracy, the haplotypecaller of gatk was initially exploited to detect indels, based on the results called by samtools. Some filters were applied to SNPs to guarantee reliability as follows: (i) minor allele frequency ≤ 0.05 and (ii) *P*‐value of Hardy‐Weinberg equilibrium test ≤ 1 × 10^−6^. A total of 202 106 SNPs (25 696 of which were identified as unreported in the pig dbSNP using a Perl script, http://hgdownload.cse.ucsc.edu/goldenPath/susScr3/database/; updated on Nov 03, 2016) and 34 415 indels were obtained. The variants were distributed on each chromosome in a relatively uniform fashion, with the exception of some isolated regions on some chromosomes (Fig. S3a and b). The resulting density distribution of variants indicated that the most variants were enriched on chromosome 12 (Fig. S4).

**Table 1:** Summary of the sample size, average genome coverage and putative population‐specific SNPs identified from sequencing data in five pig populations

Population	Distribution	*n*	Average genome coverage (%)	Average sequencing depth^a^	No. of specific SNPs^b^
Huaibei	Lianyungang, Northern Jiangsu Province	33	1.9	12.42	50
Shanzhu	Nanjing, Central Jiangsu Province	19	2.3	13.06	32
Dongchuan	Taixing, Central Jiangsu Province	9	1.4	6.64	0
Jiangquhai	Taizhou, Central Jiangsu Province	38	2.7	10.18	46
Hongdenglong	Changzhou, Southern Jiangsu Province	30	2.5	13.26	31

a The sequencing depth was estimated based on the SNP identification.

b SNPs for which one of the alleles was present in only one population (Ramos *et al*. 2011).

According to the gene annotation set extracted from the Ensembl website (ftp://ftp.ensembl.org/pub/release-78/gtf/sus_scrofa/) (Flicek *et al*. 2014), SNPs and indels were located in 9265 and 5305 genes respectively (Table S1). Furthermore, 2690 SNPs and 224 indels were mapped to exons. Out of these variants, 916 SNPs were non‐synonymous and 198 indels were frameshift mutations (Table S2). The variants that were capable of inducing protein‐coding changes were used for enrichment analysis. Bioinformatics analysis was performed using Gene Ontology (GO) terms and Kyoto Encyclopedia of Genes and Genomes (KEGG) pathways. The detailed method is shown in Appendix S1. Among the top (1%) statistically significant GO terms, genes containing SNPs were enriched in developmental process involved in reproduction (GO:0003006) and response to xenobiotic stimulus (GO:0009410) (Table S3). Chinese indigenous pigs are renowned for their reproductive traits, strong resistance to diseases, good adaptation and superior meat quality (Bosse *et al*. 2014). These observations suggest that the SNPs under investigation may have an effect on reproductive performance and disease resistance in the five pig populations. Moreover, the KEGG pathways in which genes containing the SNPs were enriched were significantly associated with the metabolism of the fatty acids (ssc01212) and biosynthesis unsaturated fatty (ssc01040), indicating that those SNPs may also be associated with meat quality in these pig populations (Table S3). Furthermore, in comparison with the results of pathway analysis, both SNPs and indels of the five populations were associated with the ECM‐receptor interaction pathway, which is consistent with the variants identified in six pig breeds of the Taihu Lake region (Wang *et al*. 2016) (Table S3). ECM‐receptor interaction is reportedly related to adipose tissue (Lee *et al*. 2013). These results indicate that these variants may be the reason for enhanced fat deposition in these Chinese indigenous pigs. Moreover, the genes containing indels were associated with the PI3K‐Akt signalling pathway, which is reportedly associated with traits of immunity and growth (Table S3) (Zhang *et al*. 2016), indicating that these indels may be associated with these two traits.

To investigate the genomic similarity and differences among the five populations, several procedures were carried out (Appendix S1). A neighbour‐joining tree was constructed, which showed that individuals from the same population were generally clustered together, with the exception of one individual from Dongchuan population (Fig. S5). The reason for this outlier may be technical or human error during sample collection. Thus, to minimise bias, this individual was excluded from further analysis. Hongdenglong and Huaibei displayed a comparatively greater distance from other populations (Fig. S5). The results of principal components analysis revealed a geographical pattern with clusters of the five populations from left to right, corresponding to northern, central and southern Jiangsu Province. Hongdenglong was isolated from the others in the first eigenvector, whereas the second eigenvector contributed mainly to the separation of Huaibei from Jiangquhai, Dongchuan and Shanzhu (Fig. 1a). The results of structure analysis showed that Huaibei and Hongdenglong formed primarily two independent populations when we hypothesized that the number of ancestral populations (*K*) equalled 2 (Fig. 1b). When *K* was increased, Jiangquhai (*K *= 3), Shanzhu (*K *= 4) and Dongchuan (*K *= 5) were progressively assigned to a distinct cluster. The results imply that Dongchuan shares a larger genetic background with the other populations, which is consistent with the results of putative population‐specific SNPs (Table 1). A previous study based on 27 microsatellite markers reported that Jiangquhai and Dongchuan form a single branch (Fan *et al*. 2002). This is because Dongchuan and Jiangquhai are located in adjacent areas, leading to a high probability of exchange of genes. Moreover, Dongchuan is located in a region within which other pig breeds are distributed. Historically, Jiangquhai in the north, Dahualian and Erhualian (Taihu pig breeds) in the south, Zhao (subpopulation of the Huai breed) in the east and Mi (Taihu pig breed) in the southwest have all contributed to the formation of Dongchuan (China National Commission of Animal Genetic Resources 2011). Some individuals among the five populations had evidence of ancestral mixture to some extent. This could be attributed to the fact that the five populations in China are all classified in conservation status categories. The various sizes of populations in conservation are generally limited. Thus, it is difficult to avoid inbreeding completely. The *F*_ST_ values were consistent with the results mentioned above (Table S4). Moreover, Huaibei and Shanzhu, which belong to the Huai breed, displayed greater differentiation than did the other populations. The reason for this inconsistency could be attributed to geographical isolation. At present, these two populations are reared in different state‐run conservation farms that are isolated from each other, with a limited number of boars.

**Figure 1:**
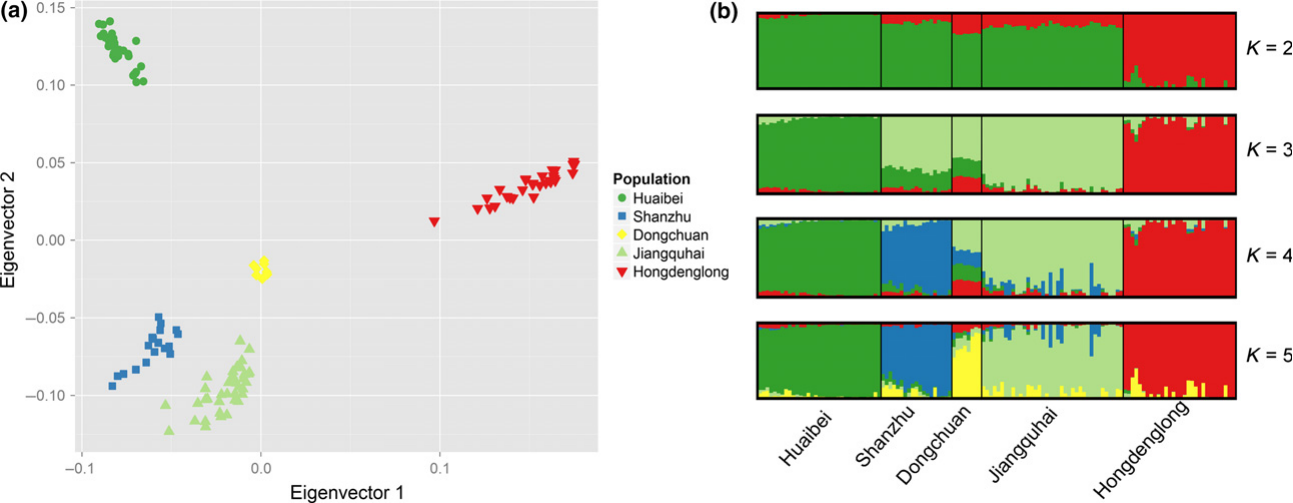
Analysis of the population structure of five pig populations. (a) Principal components analysis of five pig populations. (b) Population structure of the five tested populations as revealed by structure software. Different colours identify different clusters.

According to the latest classification of the Chinese indigenous pig breeds, Hondenglong was not considered among the animal genetic resources of China (China National Commission of Animal Genetic Resources 2011). However, in the present study, higher average *F*_ST_ values were observed in Hongdenglong for each pair‐wise comparison of the tested populations, indicating considerable differentiation between this population and others. Moreover, both the neighbour‐joining tree and principal components analysis showed a large distance between Hongdenglong and the other populations, and the results of the structure analysis demonstrated the unique genetic structure of this population.

In conclusion, our findings could provide valuable information to facilitate further exploration of the genetic mechanisms of phenotypic characters, genetic diversity and molecular evolutionary history of these five populations of Chinese indigenous pig breeds. This exploration could in turn address future breeding and conversation of biodiversity more effectively.

## Data availability

All BAM data were deposited in the National Center for Biotechnology Information (NCBI) Sequence Read Archive (SRA) under the Bioproject number PRJNA281578. The experiment numbers for the 129 pigs are SRX1739624, SRX1800626 and SRX1801076. The SRA submission number is SRP057434. The SNP and indel data have been submitted to the dbSNP of NCBI.

## Conflict of interest

The authors declare no conflict of interest.

## Authors' contributions

YP designed the study. YP and QW supervised the study. QX performed the experiments and wrote the manuscript. ZZ analyzed the data. HS, HY, MX, XL, WZ, YZ and MZ contributed to the collection of samples. All authors have read and edited the manuscript.

## Supporting information


**Figure S1** Geographic distribution of pig populations of Jiangsu Province.

**Figure S2** Average base quality scores of reads. (a) Quality distribution of each base of the raw and filtered reads from R1 (5′). (b) Quality distribution for each base of raw and filtered reads from R2 (5′). The red line represents the sequencing quality of the raw reads. The green line represents the sequencing quality of the filtered reads. (c) Distribution of the average good reads for individuals within each population.

**Figure S3** Distribution of variants across genomes located in each chromosome. (a) Distribution of SNPs located on each chromosome. (b) Distribution of indels located on each chromosome.

**Figure S4** Density distribution of variants across chromosomes.

**Figure S5** Neighbour‐joining tree of five pig populations.

**Table S1** Distribution of genetic variants detected on each chromosome.

**Table S2** Statistics of genetic variants in functional regions of genes.

**Table S3** Significant GO terms and KEGG pathways of the variants located in gene exons.

**Table S4** Genetic differentiation (*F*_ST_ values) among the tested pig populations.

**Appendix S1** Detailed information of materials and methods.
