# Supplementary material for: Genetic variation and genetic structure of five Chinese indigenous pig populations in Jiangsu Province revealed by sequencing data
Source: Anim Genet. 2017 May 22;48(5):596–9. doi: 10.1111/age.12560 (PMC5638066; doi:10.1111/age.12560)

Figure S1 Geographic distribution of pig populations of Jiangsu Province. The red line represents the boundary of Jiangsu Province, the orange points represent the Taihu pig breeds and the blue points indicate the tested pig populations.

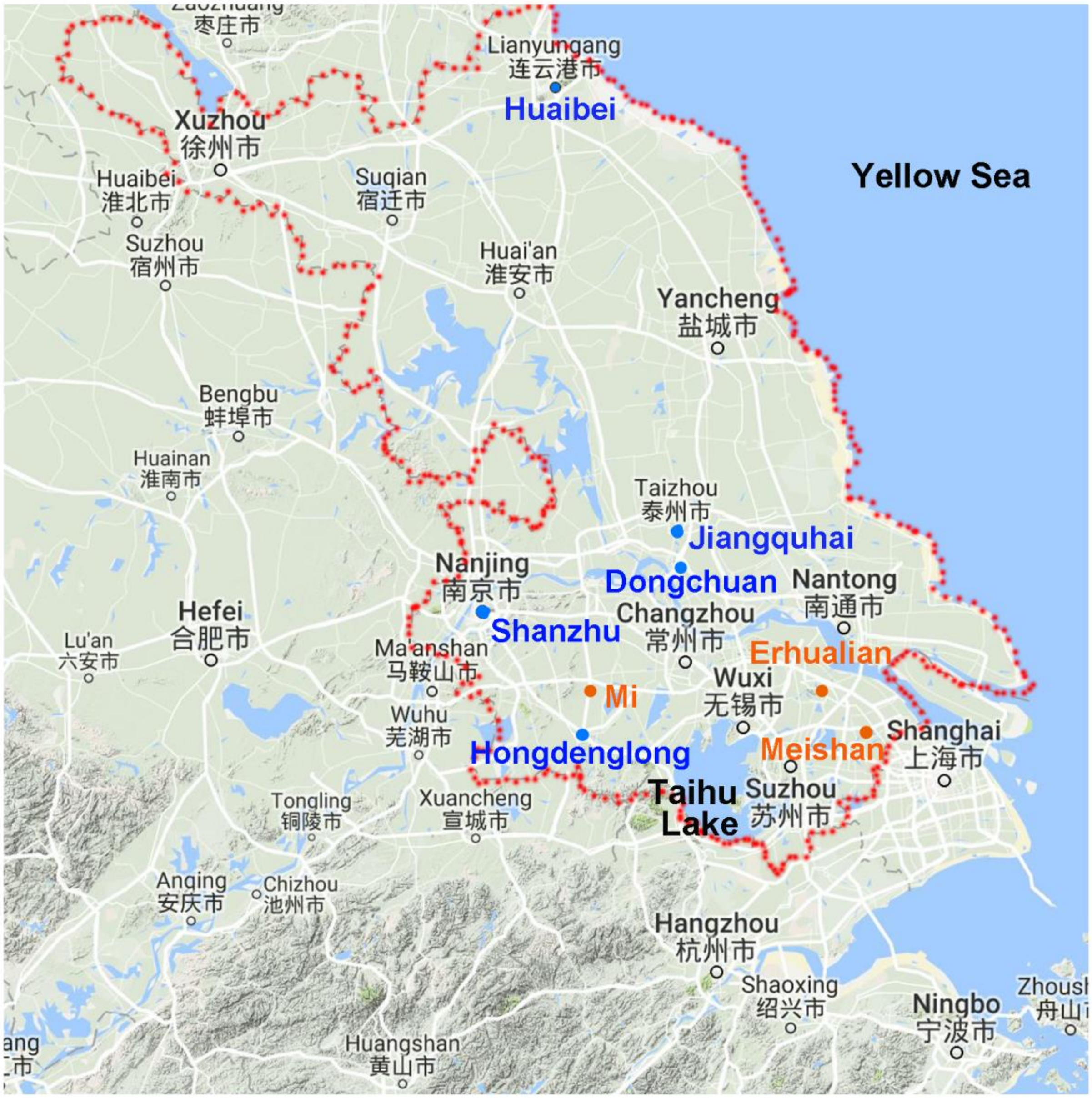

Supplement: Supplementary file 1 — Figure S1 Geographic distribution of pig populations of Jiangsu Province. [file AGE-48-596-s001.pdf]
