# Supplementary material for: Genetic variation and genetic structure of five Chinese indigenous pig populations in Jiangsu Province revealed by sequencing data
Source: Anim Genet. 2017 May 22;48(5):596–9. doi: 10.1111/age.12560 (PMC5638066; doi:10.1111/age.12560)

Figure S2 Average base quality scores of reads. (a) Quality distribution of each base of the raw and filtered reads from R1 (5'). (b) Quality distribution for each base of raw and filtered reads from R2 (5'). The red line represents the sequencing quality of the raw reads. The green line represents the sequencing quality of the filtered reads. (c) Distribution of the average good reads for individuals within each population.

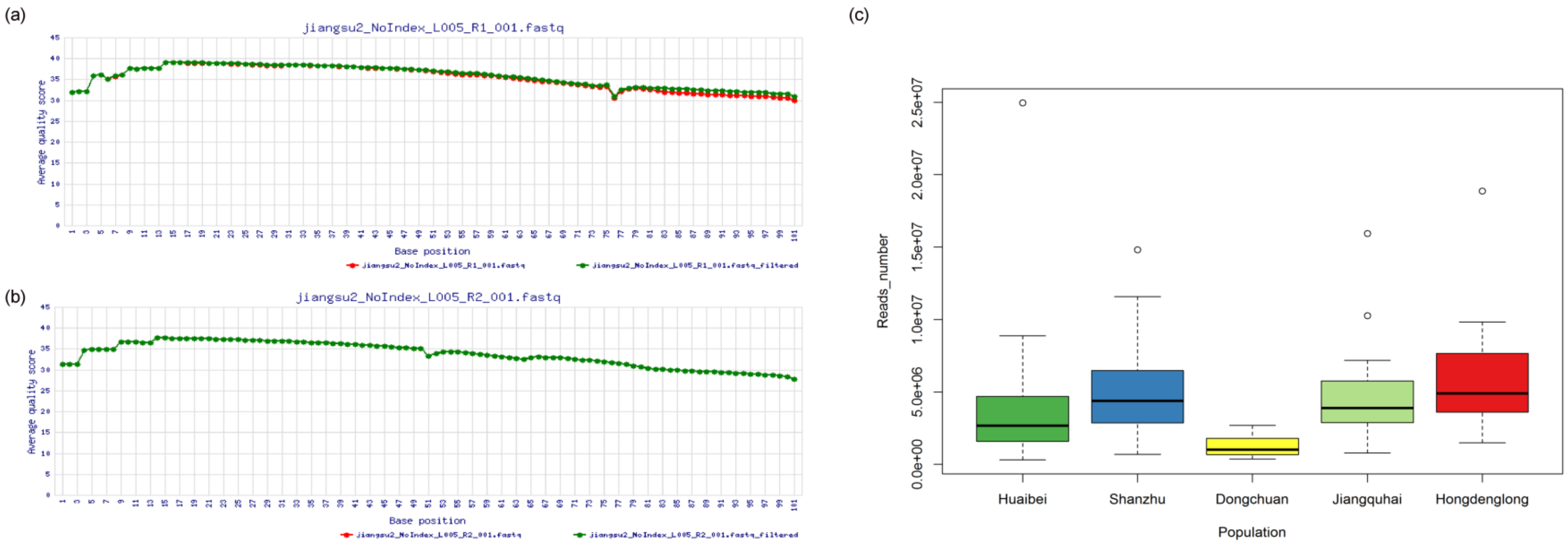

Supplement: Supplementary file 2 — Figure S2 Average base quality scores of reads. (a) Quality distribution of each base of the raw and filtered reads from R1 (5′). (b) Quality distribution for each base of raw and filtered reads from R2 (5′). The red line represents the sequencing quality of the raw reads. The green line represents the sequencing quality of the filtered reads. (c) Distribution of the average good reads for individuals within each population. [file AGE-48-596-s002.pdf]
