# Supplementary material for: Genetic variation and genetic structure of five Chinese indigenous pig populations in Jiangsu Province revealed by sequencing data
Source: Anim Genet. 2017 May 22;48(5):596–9. doi: 10.1111/age.12560 (PMC5638066; doi:10.1111/age.12560)

Figure S3 Distribution of variants across genomes located in each chromosome. (a) Distribution of SNPs located on each chromosome. (b) Distribution of indels located on each chromosome. The number of variants present in each 400-kb genome block was calculated. Deeper colour indicates a higher number of variants.

(a)

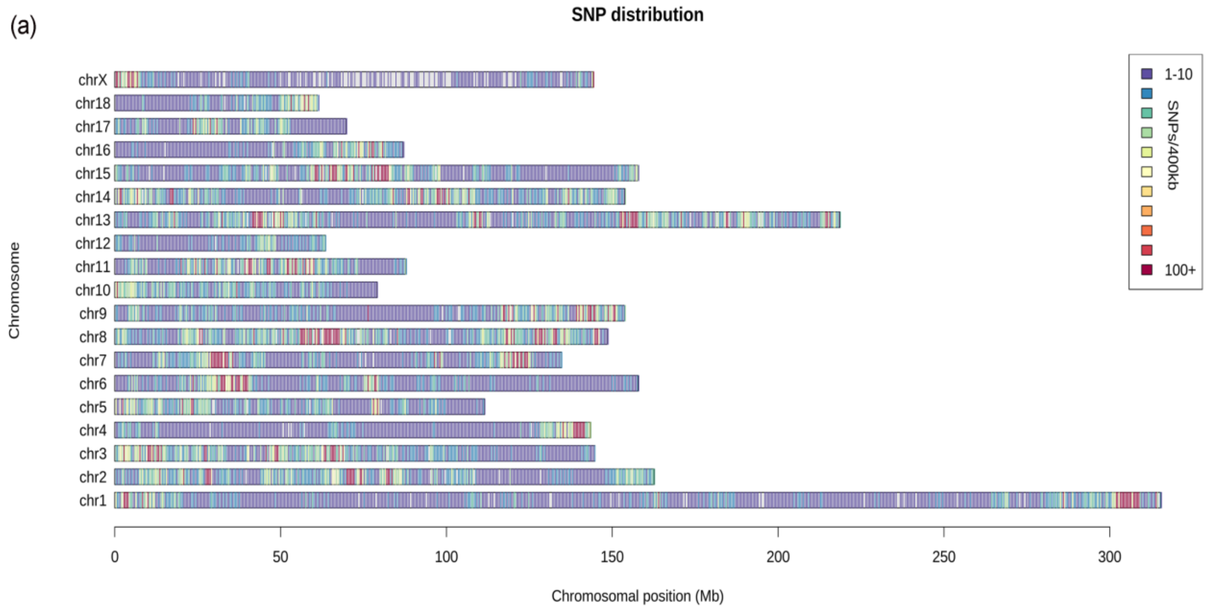

(b)

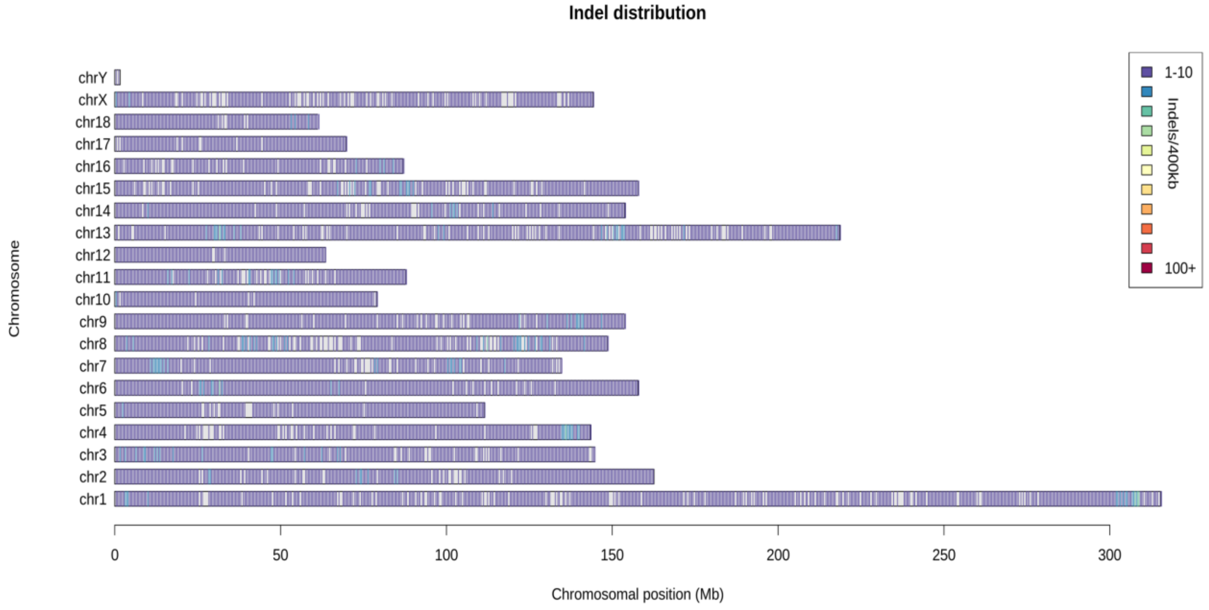

Supplement: Supplementary file 3 — Figure S3 Distribution of variants across genomes located in each chromosome. (a) Distribution of SNPs located on each chromosome. (b) Distribution of indels located on each chromosome. [file AGE-48-596-s003.pdf]
