# Supplementary figures and images for: Genetic variation and genetic structure of five Chinese indigenous pig populations in Jiangsu Province revealed by sequencing data
Source: Anim Genet. 2017 May 22;48(5):596–9. doi: 10.1111/age.12560 (PMC5638066; doi:10.1111/age.12560)

**Figure S4** Density distribution of variants across chromosomes.

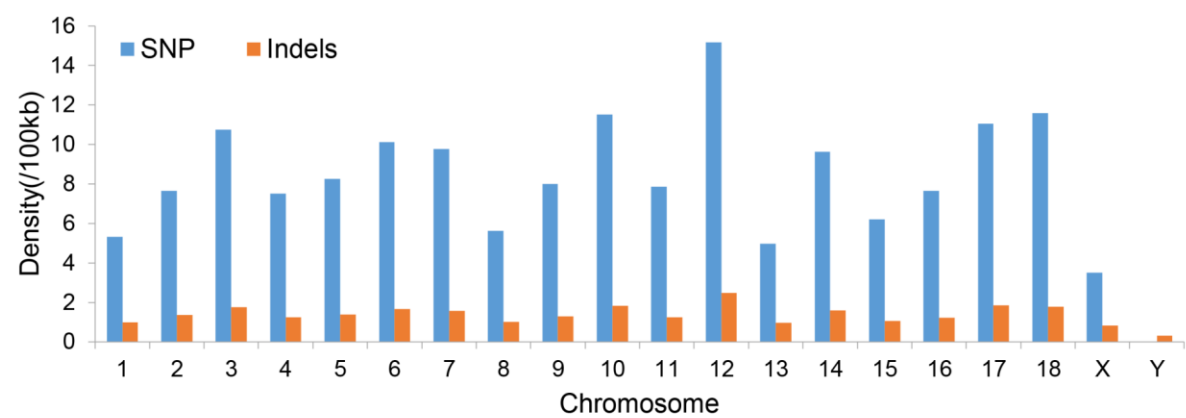

Supplement: Supplementary file 4 — Figure S4 Density distribution of variants across chromosomes. [file AGE-48-596-s004.pdf]

**Figure S5** Neighbour-joining tree of five pig populations.

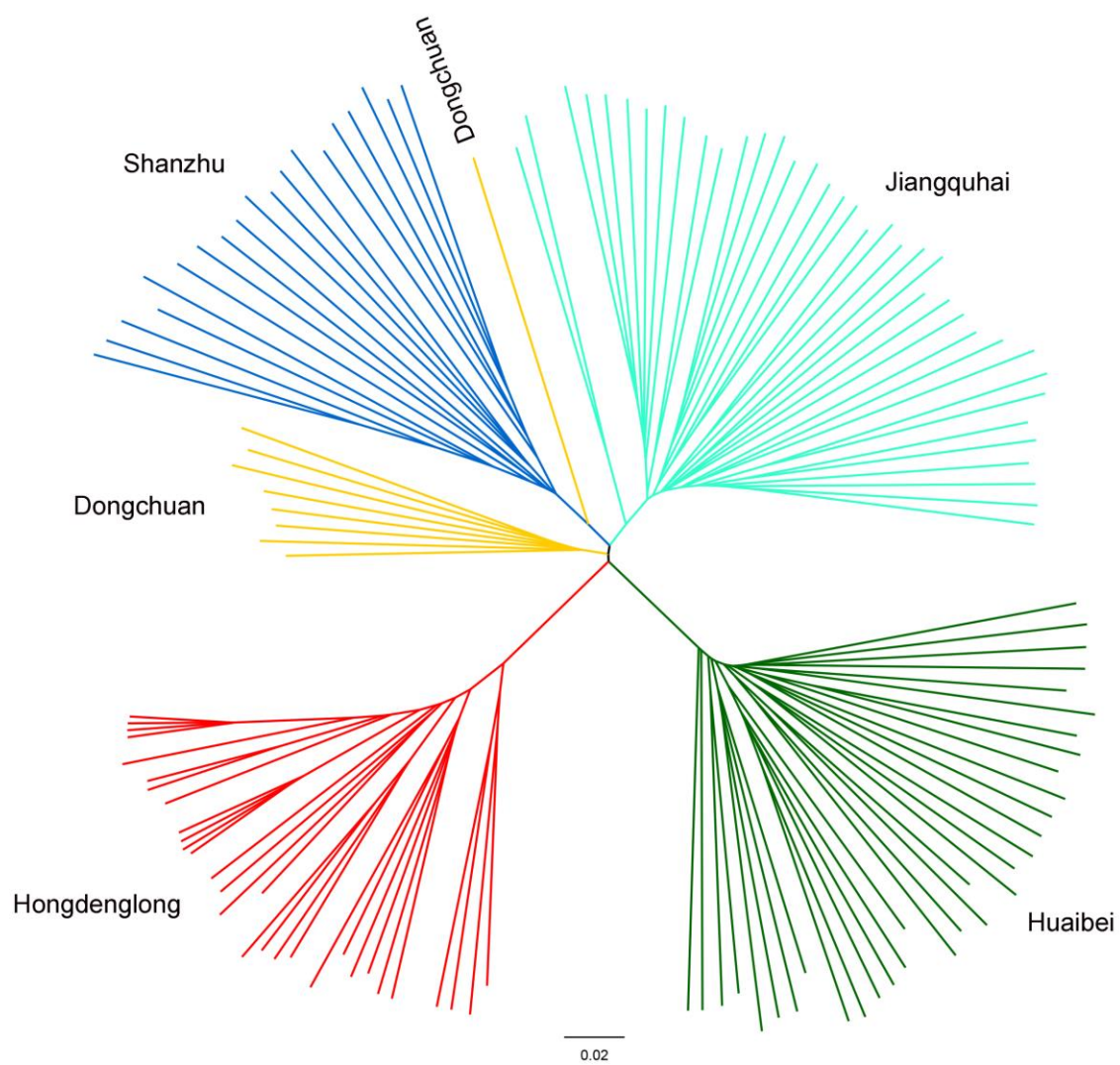

Supplement: Supplementary file 5 — Figure S5 Neighbour‐joining tree of five pig populations. [file AGE-48-596-s005.pdf]
