# Supplementary material for: Genetic variation and genetic structure of five Chinese indigenous pig populations in Jiangsu Province revealed by sequencing data
Source: Anim Genet. 2017 May 22;48(5):596–9. doi: 10.1111/age.12560 (PMC5638066; doi:10.1111/age.12560)
Supplement: Supplementary file 6 — Table S1 Distribution of genetic variants detected on each chromosome. [file AGE-48-596-s006.pdf]

Table S1 Distribution of genetic variants detected on each chromosome.

Chr	SNP			Indels			Genes
	NO.[1]	Genes	Ratio[2]	*NO.*[1]	Genes	Ratio[2]	*NO.*[3]
**1**	5762	850	38.48%	935	516	23.36%	2,209
**2**	4151	824	38.38%	662	428	19.93%	2,147
**3**	4899	672	46.54%	665	382	26.45%	1,444
**4**	2870	485	38.68%	479	263	20.97%	1,254
**5**	3540	497	42.41%	427	276	23.55%	1,172
**6**	4814	850	43.79%	780	468	24.11%	1,941
**7**	4294	590	35.74%	627	342	20.71%	1,651
**8**	2330	353	41.68%	390	202	23.85%	847
**9**	3776	529	37.07%	489	278	19.48%	1,427
**10**	2336	249	47.52%	393	162	30.92%	524
**11**	1584	206	48.82%	289	131	31.04%	422
**12**	3505	534	46.64%	389	306	26.72%	1,145
**13**	3397	592	38.59%	595	347	22.62%	1,534
**14**	5304	641	46.79%	733	366	26.72%	1,370
**15**	2703	378	39.38%	417	205	21.35%	960
**16**	1538	192	43.24%	320	109	24.55%	444
**17**	2431	322	46.80%	363	185	26.89%	688
**18**	2256	252	49.22%	316	151	29.49%	512
**X**	1482	249	22.23%	332	186	16.61%	1,120
**Y**	0	0	0.00%	2	2	15.38%	13
**Total**	62,972	9,265	44.05%	9,603	5,305	23.24%	22,824

[1] The number of identified variants within genic regions.

[2] The ratio of the number of genes containing variants accounting for the total genes within one chromosome.

[3] The number of total genes contained in the Ensembl gene database of pigs.
