# Supplementary material for: Genetic variation and genetic structure of five Chinese indigenous pig populations in Jiangsu Province revealed by sequencing data
Source: Anim Genet. 2017 May 22;48(5):596–9. doi: 10.1111/age.12560 (PMC5638066; doi:10.1111/age.12560)
Supplement: Supplementary file 7 — Table S2 Statistics of genetic variants in functional regions of genes. [file AGE-48-596-s007.pdf]

Table S2 Statistics of genetic variants in functional regions of genes.

Category	SNP	Indels
Intergenic	143,181	24,256
Exonic(Stop gain or loss)	14	5
Exonic(Non-synonymous/Frameshift)	916	198
Exonic(Synonymous/Non-frameshift)	1,760	21
Intronic	51,751	9,052
Splicing[1]	5	20
Upstream and downstream[2]	4,479	863
Total	202,106	34,415

[1] within 2 bp of a splicing junction.

[2] within a 1 kb region upstream or downstream from the transcription start site.
