# Supplementary material for: Genetic variation and genetic structure of five Chinese indigenous pig populations in Jiangsu Province revealed by sequencing data
Source: Anim Genet. 2017 May 22;48(5):596–9. doi: 10.1111/age.12560 (PMC5638066; doi:10.1111/age.12560)
Supplement: Supplementary file 9 — Table S4 Genetic differentiation (F ST values) among the tested pig populations. [file AGE-48-596-s009.pdf]

Table S4 Genetic differentiation (F_{ST} values) among the tested pig populations.

Population	Shanzhu	Dongchuan	Jiangquhai	Hongdenglong
Huaibei	0.157	0.128	0.160	0.250
Shanzhu		0.101	0.110	0.237
Dongchuan			0.068	0.167
Jiangquhai				0.196
