# Supplementary material for: Genetic variation and genetic structure of five Chinese indigenous pig populations in Jiangsu Province revealed by sequencing data
Source: Anim Genet. 2017 May 22;48(5):596–9. doi: 10.1111/age.12560 (PMC5638066; doi:10.1111/age.12560)
Supplement: Supplementary file 10 — Appendix S1 Detailed information of materials and methods. [file AGE-48-596-s010.pdf]

Appendix S1 Detailed information of materials and methods.

Sample collection and DNA library sequencing

Five populations were evaluated in the present study: two breeds (Jiangquhai, n=38 and Dongchuan, n=9); two subpopulations of the Huai breed (Huaibei, n=33 and Shanzhu, n=19); and one population that has not been previously recorded in the animal genetic resources of China (China National Commission of Animal Genetic Resources 2011) (Hongdenglong, n=30). All samples were collected from conservation pig farms in Jiangsu Province, China. DNA was extracted from ear tissue, using a Lifefeng blood and tissue extraction kit (Lifefeng Biotech (Shanghai) Co., Ltd, China). The DNA libraries (insert size varied from 200 to 300 bp) were constructed using the GGRS protocol (http://klab.sjtu.edu.cn/GGRS/) (Chen *et al.* 2013). High quality genomic DNA from all samples was digested with a restriction enzyme (*Ava*Ⅱ) and then ligated with an adapter barcode. Each sample was ligated with a unique adapter barcode. The samples were then pooled and enriched through PCR, to construct the sequencing library. DNA libraries were sequenced with the Illumina HiSeq 2500 (100 paired-end) sequencer. Quality control (QC) procedures of the raw reads were carried out using the NGS QC Toolkit (v2.3) and the filtration criteria referred to by Chen *et al.* (2013). Filtered reads from all individuals were aligned to the pig reference genome (SGSC Sscrofa10.2, http://hgdownload.soe.ucsc.edu/goldenPath/susScr3/bigZips/) by the Burrows-Wheeler Aligner (BWA, version 0.7.5) (Li & Durbin 2009). Alignment results were trimmed by removing mapping quality scores less than 20. The remaining reads were defined as good reads.

Function annotation

Annotation was performed based on to the Pig Reference Genome (*Sscrofa10.2*) using the ANNOVAR software package (version: 2016 Feb 01) (Wang *et al.* 2010; Yang & Wang 2015). Based on genome annotation, the genes containing the variants that can induce amino acid changes (non-synonymous SNPs and frameshift Indels) were extracted for enrichment analysis in Gene Ontology (GO) terms (Ashburner *et al.* 2000) and Kyoto Encyclopedia of Genes and Genomes (KEGG) pathways (Kanehisa & Goto 2000). GO analysis was implemented using the GOSeq R package (Young *et al.* 2010), in which gene length bias was

adjusted. KEGG enrichment analysis was analysed using the KEGG Orthology Based Annotation System (KOBAS) 2.0 server (Pie *et al.* 2011). The top enriched GO terms (1%) and statistically significant KEGG pathways (p-value less than 0.05) were used for further analysis.

Analysis of genetic structure and population differentiation

Various methods were used to provide an assessment of the genetic structure of the tested populations. A neighbour-joining (NJ) tree was first constructed using the MEGA v6.06 software (Tamura *et al.* 2013), to reflect topological relationships among all individuals based on the identity-by-state (IBS) distance matrix data, which was calculated using the PLINK v1.07 software (Purcell *et al.* 2007). Principal component analysis (PCA) was then performed using the Genome-Wide Complex Trait Analysis (GCTA) software (version 1.24) (Yang *et al.* 2011). The STRUCTURE (v2.3.4) software (Greenbaum *et al.* 2016) was carried out to assess further the underlying ancestry proportions among individuals. Visualization of the results was plotted with the DISTRUCT (v1.1) program (Rosenberg, 2004).

To investigate the extent of differentiation among the tested populations, an unbiased genetic differentiation estimate of the fixation index (F_{ST}) was calculated using all available SNPs (Balloux & Lugon-Moulin 2002) according to the method described by Weir & Cockerham (1984).

References

Ashburner M., Ball C.A., Blake J.A., Botstein D., Butler H., Cherry J.M., Davis A.P., Dolinski K., Dwight S.S. & Eppig J.T. (2000) Gene Ontology: tool for the unification of biology. *Nature genetics* **25**, 25-9.

Balloux F. & Lugon-Moulin N. (2002) The estimation of population differentiation with microsatellite markers. *Molecular Ecology* **11**, 155-65.

Chen Q., Ma Y., Yang Y., Chen Z., Liao R., Xie X., Wang Z., He P., Tu Y. & Zhang X. (2013) Genotyping by genome reducing and sequencing for outbred animals. *PLoS One* **8**, e67500.

Flicek P., Amode M.R., Barrell D., Beal K., Billis K., Brent S., Carvalho-Silva D., Clapham

P., Coates G. & Fitzgerald S. (2013) Ensembl 2014. *Nucleic acids research*, gkt1196.

Greenbaum G., Templeton A.R. & Bar-David S. (2016) Inference and Analysis of Population Structure Using Genetic Data and Network Theory. *Genetics* **202**, 1299-312.

Kanehisa M. & Goto S. (2000) KEGG: kyoto encyclopedia of genes and genomes. *Nucleic Acids Res* **28**, 27-30.

Huang D.W., Sherman B.T. & Lempicki R.A. (2009) Systematic and integrative analysis of large gene lists using DAVID bioinformatics resources. *Nature protocols* **4**, 44-57.

Li H. & Durbin R. (2009) Fast and accurate short read alignment with Burrows–Wheeler transform. *Bioinformatics* **25**, 1754-60.

Purcell S., Neale B., Todd-Brown K., Thomas L., Ferreira M.A., Bender D., Maller J., Sklar P., de Bakker P.I., Daly M.J. & Sham P.C. (2007) PLINK: a tool set for whole-genome association and population-based linkage analyses. *American journal of human genetics* **81**, 559-75.

Rosenberg N. A. (2004) Distruct: a program for the graphical display of population structure. Mol. Ecol. Notes **4**, 137–138.

Tamura K., Stecher G., Peterson D., Filipski A. & Kumar S. (2013) MEGA6: molecular evolutionary genetics analysis version 6.0. *Molecular biology and evolution*, mst197.

Wang K., Li M. & Hakonarson H. (2010) ANNOVAR: functional annotation of genetic variants from high-throughput sequencing data. *Nucleic Acids Res* **38**, e164.

Weir BS and Cockerham CC. (1984) Estimating F-Statistics for the Analysis of Population Structure. *Evolution* **38**, 1358-1370.

Xie C., Mao X., Huang J., Ding Y., Wu J., Dong S., Kong L., Gao G., Li C.Y. & Wei L. (2011) KOBAS 2.0: a web server for annotation and identification of enriched pathways and diseases. *Nucleic Acids Res* **39**, W316-22.

Yang H. & Wang K. (2015) Genomic variant annotation and prioritization with ANNOVAR and wANNOVAR. *Nat Protoc* **10**, 1556-66.

Yang J., Lee S.H., Goddard M.E. & Visscher P.M. (2011) GCTA: a tool for genome-wide complex trait analysis. *American journal of human genetics* **88**, 76-82.

Young M.D., Wakefield M.J., Smyth G.K. & Oshlack A. (2010) Gene ontology analysis for

RNA-seq: accounting for selection bias. *Genome biol* **11**, R14.
